# Modulating the extension of thyroidectomy in patients with papillary thyroid carcinoma pre-operatively eligible for lobectomy: reliability of ipsilateral central neck dissection

**DOI:** 10.1007/s12020-020-02456-5

**Published:** 2020-08-20

**Authors:** M. Raffaelli, C. De Crea, L. Sessa, S. E. Tempera, G. Fadda, A. Pontecorvi, R. Bellantone

**Affiliations:** 1 U.O.C. Chirurgia Endocrina e Metabolica, Fondazione Policlinico Universitario Agostino Gemelli IRCCS, Rome, Italy; 2 Dipartimento Universitario di Medicina e Chirurgia Traslazionale, Università Cattolica del Sacro Cuore, Rome, Italy; 3 U.O.C. di Chirurgia Generale - Ospedale Fatebenefratelli, Milan, Italy; 4 U.O.C. Anatomia Patologica, Fondazione Policlinico Universitario Agostino Gemelli IRCCS, Rome, Italy; 5 U.O.C. Endocrinologia e Diabetologia, Fondazione Policlinico Universitario Agostino Gemelli IRCCS, Rome, Italy

**Keywords:** Papillary thyroid carcinoma, Central neck dissection, Thyroid lobectomy, Frozen section examination, Personalized medicine, Personalized surgery

## Abstract

**Purpose:**

Pre-operative work-up and macroscopic intraoperative inspection could overlook occult central neck nodal metastases in patients with papillary thyroid carcinoma (PTC). An occult N1a status is able to change the initial risk stratification in small, clinically unifocal PTC potentially scheduled for thyroid lobectomy (TL) making total thyroidectomy (TT) the preferable option. We aimed to verified the reliability of an intraoperative management protocol based on frozen section examination (FSE) of ipsilateral central neck nodes (IpsiCND) to identify, among patients scheduled for TL, those who could benefit of a more extensive surgical resection (TT plus bilateral central neck dissection -CND-).

**Methods:**

Thirty PTC patients preoperatively classified as T1N0 underwent TL plus IpsiCND-FSE (TL-group). In case of positive FSE, TT plus bilateral CND was accomplished during the same surgical procedure. A comparative analysis was performed between TL-group and a control group (C-group), who underwent TT plus IpsiCND-FSE, matched by a propensity score analysis.

**Results:**

Nodal metastases (>2 mm) were found at final histology in 5/30 patients in the TL-group and in 6/30 in the C-group (*p* = 1.00). Micrometastases (≤2 mm) were retrieved in 5/30 TL-group patients and in 4/30 C-group patients (*p* = 1.00). Final histology staged as pN1a 10 (33.3%) patients for each group. FSE correctly identified five patients with occult nodal metastases >2 mm (16.6%) in TL-group, who underwent TT plus bilateral CND during the same surgical procedure. No permanent complications occurred. At a mean follow-up of 22.2 months, no local and/or nodal recurrence were observed.

**Conclusions:**

Intraoperative assessment of N status obtained with IpsiCND plus FSE allows for an accurate risk stratification. IpsiCND plus FSE real time modulated thyroidectomy seems a safe and effective surgical strategy reducing the need of a subsequent completion surgery and, theoretically, the risk of local recurrence.

## Introduction

Current guidelines endorse thyroid lobectomy (TL) as initial surgical approach for low risk, small (T1) clinically unifocal, intrathyroidal, No papillary thyroid carcinoma (PTC) [1–3]. Several evidences support this approach as no benefits of total thyroidectomy (TT) over TL are demonstrated in terms of disease-specific overall survival [4–8].

On the other hand, overall survival alone could not be the best indicator of adequacy of initial surgical treatment. Indeed, it is well known that several PTC patients could experience recurrence and require additional/s surgical treatments [9–11]. PTC recurrences are associated to a reduced quality of life and often to technical demanding surgical re-exploration [12].

From this point of view the choice of initial surgical treatment plays an important role and it should not be based on tumor size alone [13–15].

Prior head and neck irradiation, familial thyroid carcinoma, multifocality, extrathyroidal extension and central and/or lateral lymph node neck metastases are considered high-risk features requiring more extended surgical resections (i.e., TT with/without lymph node dissection).

Current guidelines focus on risk stratification of PTC patients to modulate treatment strategy [1–3]. Unfortunately, some of the above mentioned risk factors are available only after final histological reports [1–3, 16]. Furthermore, there are evidences demonstrating that up to 40% of patients may be upstaged to a higher risk category after limited thyroid resection (i.e., TL) for PTC [13].

An accurate preoperative work up could led to identify some suspicious characteristics (e.g., macroscopic evidence of multifocality or extrathyroidal extension; evidence of lateral neck lymph node metastases) [11, 14, 15, 17], but it is well known that it is hard to reliably define the central neck nodal status pre- and even intra-operatively [18–20].

In order to assess the central neck lymph node status and to modulate the extension of central neck dissection (CND), reducing postoperative complications related to prophylactic bilateral CND, we proposed and validated frozen section examination (FSE) on the ipsilateral central neck lymph nodes (IpsiCND) for selected patients with clinical unifocal cN0 PTC.

We supposed that FSE evaluation of IpsiCND could be useful to modulate the extension of surgical procedure in PTC patients eligible for TL. In the case occult nodal metastases are found at FSE, TT and bilateral central compartment clearance become mandatory [21–24]. We adopted this surgical approach to tailor the extension of thyroidectomy and CND in clinically unifocal, small (T1), cN0 PTC. In this case-control study, we aimed to evaluate the results of such approach in the surgical management of PTC patients initially scheduled for TL.

## Materials and methods

### Patients’ population

Among 952 patients who underwent initial surgery for PTC between October 2014 and June 2017, 30 consecutive consenting patients underwent TL plus IpsiCND-FSE (TL-group) for clinically T1, unifocal, intrathyroidal, cN0 preoperative known PTC. Every PTC patient eligible for TL was informed regarding risks and benefits of TL and TT, based on available guidelines. Exclusion criteria for TL plus IpsiCND-FSE were: prior head and neck irradiation, family history of thyroid carcinoma, clinical evidence of multifocality and/or extrathyroidal extension, evidence of central and/or lateral lymph node neck metastases, previous surgical treatment for PTC.

A control group (C-group) of 30 patients with the same preoperative known characteristics, matched for age, sex, and tumor size by propensity score analysis, was selected among those patients who underwent TT plus IpsiCND-FSE as primary surgery for clinically T1, unifocal, intrathyroidal, cN0 PTC during the same study period.

### Definitions

PTCs were defined as clinically unifocal, intrathyroidal, and cN0 in the absence of any preoperative (i.e., clinical and ultrasound examination) or intraoperative evidence of multifocal disease, extracapsular invasion, or lymph node involvement, respectively.

TL was defined as extracapsular removal of one thyroid lobe with the isthmus. TT was defined as total bilateral extracapsular thyroid removal. Ipsilateral central compartment neck dissection included pre-laryngeal, pretracheal, and the paratracheal nodal basins on the side of the tumor [25]. Bilateral central compartment neck dissection included the removal of pre-laryngeal, pretracheal, and both the right and left paratracheal nodal basins [25]. All the surgical procedures were performed by an experienced endocrine surgeon or by a resident operating under supervision. Pathological tumor staging was defined in accordance with the 2010 7th edition of the American Joint Committee on Cancer pTNM staging system [26]. Occult lymph node metastases were defined as micrometastases if they measured ≤2 mm at final histology (FH) [27].

Local recurrent disease was defined as clinically detectable disease in the thyroid bed. Nodal recurrent disease was defined as clinically detectable disease in the central and/or lateral compartment lymph nodes. Distant metastases (disease outside the thyroid bed and cervical lymph node) were identified in the presence of an elevated serum thyroglobulin (sTg) and/or sites of uptake on postoperative radioactive iodine scan, eventually confirmed by the means of radiological studies.

### Study design

IpsiCND-FSE was performed as the first step of the procedure in both group. TL or TT was accomplished while waiting for the FSE results. Contralateral paratracheal nodes were removed as the last step of the procedure when ipsilateral node metastases were found on FSE for both TL- and C-group. In addition concomitant completion thyroidectomy was accomplished in the TL-group after IpsiCND-FSE positive for metastases.

The data of the two groups of patients were retrospectively obtained from an institutional surgical database in which the medical records of all the patients operated for a thyroid carcinoma were prospectively collected since January 2008. For the purpose of the present study, the following parameters were registered in a specifically designed database (Microsoft Excel^®^, Microsoft Corporation, Redmond, WA, USA): age, sex, tumor size, surgical procedure, operative time, postoperative complications, hospital stay, pathological diagnosis, and follow-up evaluation. Follow-up evaluation was obtained by outpatient consultation and/or telephone contact. A comparative analysis between the two groups concerning the registered parameters was performed.

### Study end points

The primary end point was to verify if IpsiCND-FSE in patient scheduled for TL is reliable for intraoperatively identifying patients who would benefit from TT + bilateral CND. Secondary end point was to compare the two groups in terms of postoperative outcome (i.e., complication rate, upstaging rate).

### Postoperative management and follow-up evaluation

The protocol for the postoperative management and follow-up evaluation of patients with PTC has been previously described [17, 21, 28–31]. Patients of TL-group underwent completion thyroidectomy with/without completion CND after FH based on postoperative tumor risk stratification and patient’s preference [1].

Ultrasound (US) neck scan 3–6 months after surgery was performed in patients who underwent TL; sTg and anti-Tg antibody measurements and an US neck scan 3–6 months after surgery was performed in patients who underwent TT. This was the only follow-up protocol adopted for patients with pT1 PTC ≤ 1.0 cm, in the absence of lymph node metastases and multifocality. The remaining patients underwent nuclear medicine evaluation.

Among patients who underwent TT, 131-I ablation (RAI) was performed on the basis of stage and risk factors, according to the American Thyroid Association Guidelines [1]. Patients were evaluated by 131-I diagnostic whole body scan (DxWBS) and TSH-stimulated sTg. TSH-stimulated sTg were obtained prior to RAI, when applicable. All the high-risk patients underwent RAI. TSH-stimulated sTg levels obtained prior to RAI and post therapy whole body scan (TxWBS) were evaluated in this group of patients.

In the subset of patients undergoing nuclear medicine evaluation, the completeness of the surgical resection was determined by neck US scan and TSH-stimulated sTg levels. Patients who did not receive RAI underwent long-term follow-up at 6–12 month intervals, at which time, an US scan was performed and sTg levels were measured while the patients were on LT4. Patients with TxWBS that revealed no thyroid remnants or metastases were also placed on this follow-up protocol. Where TxWBS revealed thyroid remnants or metastases, these patients underwent new DxWBS and sTg off LT4 6–12-months after previous RAI.

### Statistical analysis

Statistical analysis, including propensity score matching, was performed using a commercially available software package (SPSS 22.0 for Windows^®^ SPSS Inc., Chicago, IL, USA). The patients were divided in two different groups, according to surgical procedures (TL + IpsiCND-FSE or TT + IpsiCND-FSE). The surgical procedures (TL + IpsiCND-FSE or TT + IpsiCND-FSE) was entered into the regression model of the propensity scores as the dependent variable. Matching of the propensity scores was obtained with the “1:1 nearest neighbor” matching method (discard = both groups, caliper = 0.2). The *χ*^2^ test was used for categorical variables, and the one-tailed *t*-test was used for continuous variables. A *P* value < 0.05 was considered significant.

## Results

Both groups included 8 males and 22 females with a mean age of 40.8 ± 9.9 years (range 20–56) in TL-group and 37.9 ± 13.8 years (range 19–67) in the C-group. The two groups were well matched for age (*t*-test, *p* = 0.35), sex (*χ*^2^ test, *p* = 0.77) and preoperative tumor size (*t*-test, *p* = 0.16). Patients’ characteristics of both groups are reported in Table 1.

**Table 1:** Demographic, clinical, operative, pathological and follow-up characteristics of the included patients

	TL-group	C-group	*P* value
Patients	30	30	–
Age (±SD^a^) (range) years	40.8 ± 9.9 (20–56)	37.9 ± 13.8 (19–67)	0.35^b^
Male/female	8/22	8/22	0.77^c^
Mean preoperative tumor size (±SD^a^) (range) mm	7.4 ± 2.1 (4–11)	8.2 ± 2.3 (4–15)	0.16^b^
Mean operative time (±SD^a^) (range) min	58.7 ± 19.4 (30–120)	68.9 ± 21.2 (35–120)	0.06^b^
Frozen section examination positive for metastases y/n	5/25	6/24	1.00^c^
CC^e^ lymph nodes examined at frozen section (±SD^a^) (range)	4.5 ± 2.1 (3–12)	5.3 ± 3.3 (3–19)	0.25^b^
Mean hospital stay (±SD^a^) (range) days	1.8 ± 0.8 (1–4)	2.1 ± 3.4 (2–4)	0.64^b^
Mean tumor size at FH^d^ (±SD^a^) (range) mm	8.1 ± 2.5 (5–12)	9.2 ± 3.2 (6–15)	0.14^b^
pT stage T1/T2/T3/T4	25/-/5/-	24/-/6/-	1.00^c^
Multifocal disease y/n	11/19	17/13	0.17^c^
Extracapsular invasion y/n	5/25	6/24	1.00^c^
Vascular invasion y/n	0/30	1/29	1.00^c^
Histology subtypes PTC – Classic/follicular/other	26/1/3	26/0/4	0.57^c^
pN stage N0/N1a	20/10	20/10	0.78^c^
Overall removed CC^e^ lymph nodes at FH^d^ (±SD^a^) (range)	4.2 ± 2.4 (3–9)	4.9 ± 3.6 (3–10)	0.38^b^
Lymph node metastases ≤2 mm/>2 mm	5/5	4/6	1.00^c^
Transient hypocalcemia y/n	6/7^f^	15/15	0.92^c^
Definitive hypoparathyroidism y/n^f^	0/13^f^	0/30	–
Transient laryngeal nerve palsy y/n	1/29	0/30	1.00^c^
Definitive laryngeal nerve palsy y/n	0/30	0/30	–
Mean follow-up time (±SD^a^) (range) months	19.6 ± 10.0 (8–42)	24.8 ± 10.7 (8–43)	0.06^b^
131-I Ablation y/n	13/17	12/18	1.00^c^
Mean TSH-stimulated serum thyroglobulin (±SD^a^) (range) ng/ml^g^	1.6 ± 2.9 (0.2–2.7)	3.5 ± 4.8 (0.2–14.3)	0.07^b^
Recurrence	0	0	–

^a^SD standard deviation

^b^*t*-test was used

^c^*χ*^2^ test was used

^d^FH: Final Histology

^e^CC: Central Compartment

^f^Available for 13/30 TL-Group patients who underwent total thyroidectomy plus bilateral CND, intraoperatively after positive frozen section examination or after FH

^g^Available for 13 TL-Group patients and 12 C-Group patients who underwent 131I ablation

FSE correctly identified all the patients with metastases >2 mm, five in TL-group (16.6%) and six in the C-group (20.0%), respectively (*χ*^2^ test, *p* = 1.00). These patients underwent completion thyroidectomy and bilateral CND in TL-group and completion bilateral CND in C-group, respectively, during the same surgical procedure.

Mean operative time was 58.7 ± 19.4 min (range 30–120) in the TL-group and 68.9 ± 21.2 min (range 35–120) in the C-group (*t*-test, *p* = 0.06).

No statistically significant difference was found between the two groups in terms of mean hospital stay, 1.8 ± 0.8 days (range 1–4) in the TL-group and 2.1 ± 3.4 days (range 2–4) in the C-group (*t*-test, *p* = 0.64) [Table 1].

At FH mean tumor size was 8.1 ± 2.5 mm (range 5–12) in the TL-group and 9.2 ± 3.2 mm (range 6–15) in the C-group (*t*-test, *p* = 0.14). FH showed 25 pT1 (9 multifocal) and 5 pT3 (2 multifocal) in the TL-group and 24 pT1 (15 multifocal) and 6 pT3 (2 multifocal) in the C-group.

Overall, at FH 10 patients per group (33.3%) were staged pN1a. Micrometastases (≤2 mm) not recognized at FSE were observed in 5 patients (20.0%) in the TL-group and in four patients (13.3%) in the C-group (*χ*^2^ test, *p* = 1.00).

The mean overall number of removed central neck nodes at FH was similar between the two groups: 6.1 ± 4.0 (range 3–17) in the TL-group vs. 8.2 ± 6.4 (range 3–27) in the C-group (*t*-test, *p* = 0.13). The mean number of central neck nodes sent for FSE was similar between the two groups: 4.2 ± 2.4 (range 3–9) in TL-Group vs. 4.9 ± 3.6 (range 3–10) - *t*-test, *p* = 0.38 [Table 1].

Eight patients in TL-group underwent completion thyroidectomy + completion CND after FH, seven due to multifocal disease (five of these patients had concomitant micromestastes also -≤2 mm- at FH), one due to extracapsular invasion. After reoperation FH showed multifocal bilateral PTC in one case and no pathologic findings in the remaining seven cases. Overall no statistically significant difference was found between the two groups in terms of complication rate. Transient hypocalcaemia was observed in 6/13 patients of the TL-group who underwent TT plus bilateral CND, intraoperatively after positive IpsiCND-FSE or after FH, and in 15/30 patients in the C-group (*χ*^2^ test, *p* = 0.92). A unilateral transient inferior laryngeal nerve injury was reported in one patient in TL-group (*χ*^2^ test, *p* = 1.00). No other complications were registered. In particular, no definitive recurrent nerve palsy and no definitive hypoparathyroidism were observed [Table 1].

Follow-up was completed in all the included patients. The mean follow-up period was 19.6 ± 10.0 months (range 8–42) in the TL-group vs. 24.8 ± 10.7 months (range 8–43) in the C-group (*t*-test, *p* = 0.06).

Among TL-group patients no recurrent disease was observed at US scan in 17 patients (16 pT1 and 1 pT1m) in whom only TL and IpsiCND-FSE was performed. RAI was performed in the remaining 13 TL-group patients (2 pT1mN0, 6 pT1mN1a, 1 pT3N0, 2 pT3N1a, and 2 pT3mN1a) who underwent intraoperative completion thyroidectomy and bilateral CND after positive FSE (5 cases) or reoperation after FH (8 cases). In these patients the mean pre-ablation TSH-stimulated sTg was 1.6 ± 2.9 ng/ml (range 0.2–2.7) and no residual or recurrent disease was observed at US scan and DxWBS or TxWBS.

Among C-group, in 18 patients (9 pT1N0, 5 pT1mN0, and 4 pT3N0) follow-up evaluation included basal sTg measurements and neck US scan. Mean sTg level was 0.2 ± 0.3 ng/ml (range 0.0–0.6) and the US scan showed no thyroid remnants or lymph node involvement. The remaining 12 patients (10 pT1mN1a and 2 pT3mN0) underwent RAI. The mean postoperative TSH-stimulated sTg was 3.5 ± 4.8 ng/ml (range 0.2–14.3) and no residual ore recurrent disease was observed at US scan and DxWBS or TxWBS [Table 1].

## Discussion

The results of the present study demonstrate our initial hypothesis that FSE of the ipsilateral central neck nodes in patients with PTC clinically eligible for TL is able to intraoperatively identify those patients who require TT and CND because of occult lymph node metastases larger than 2.0 mm. In other word, FSE is able to reliably risk stratify PTC patients regarding the nodal status (N), allowing to modulate and personalize the extension of the surgical resection, based on objective parameters.

TL is considered the most suitable approach in patients with small (T1), intrathyroidal, clinically unifocal, N0 PTC in the absence of high-risk features, which encompass the need for a more aggressive approach (TT plus bilateral CND) [1–3]. Of note at least some of high-risk characteristics may be diagnosed only after FH [9, 11, 13, 32]. This is one of the main reason maintaining the debate regarding the optimal surgical treatment strategy in PTC. No benefit in terms of disease-specific overall survival of TT over TL are reported in selected PTC patients [4, 5]. In spite TT has no clear benefit over TL in terms of overall survival, the increased risk of recurrence following limited resection may imply the need for challenging and risky reoperative surgery [33]. Consequently, in our opinion the choice of initial surgical treatment plays a key role in the treatment of PTC patients and should not rely on tumor size alone [34]. Interestingly, in retrospective series up to 59% of PTC preoperatively classified as low risk were upstaged to a higher risk category after FH [32–34].

Despite an adequate preoperative study generally is able to identify clinical evidence of extrathyroidal extension and/or multifocality and/or lateral neck lymph nodes involvement in PTC patients, the N stage of the central neck is quite hard to define both preoperatively and intraoperatively since occult central nodal metastases occurred in 40–80% of PTC patients [11, 14–20].

In order to overcome the limitation of preoperative work up and intraoperative evaluation in determining the central neck nodal status and to modulate the extension of CND, reducing postoperative complications related to bilateral prophylactic CND, we proposed and validated FSE on IpsiCND for selected clinically unifocal cN0 PTC patients. IpsiCND seems ad adequate option in selected patients since isolated contralateral central compartment nodal metastases are exceptionally reported (0–3.6%) [20, 22]. We have found that FSE has a sensitivity, specificity, and overall accuracy of 80.7%, 100%, and 90%, respectively, in detecting occult ipsilateral central neck metastases [20, 22]. We supposed that this approach could intraoperative identify pT1 PTC patients, initially scheduled for TL, with occult central neck nodal metastases requiring TT and bilateral CND. We designed this case-control study to investigate about the use of FSE on IpsiCND in patients in which preoperative work up have suggested TL as preferable initial surgical treatment. Based on the evidences that recently led to the drawing of the most recent ATA guidelines, we proposed TL plus IpsiCND-FSE to all the patients with pre-operative diagnosis of T1 PTC without known high-risk features (prior head and neck irradiation, familial thyroid carcinoma, multifocality, extrathyroidal extension, and central and/or lateral lymph node neck metastases). Patients were informed regarding risks and benefits of both TL and TT, suggesting TL as initial surgical treatment endorsed by the most recent scientific evidences. However, many patients are still reluctant to undergo a conservative surgery, based on historical perspective and willing to avoid any risk, even if minimal, of reoperation for completion. This is the reason why, during the study period only a minority of eligible patients accepted to be included in the TL-group, and most of them only after the publication of the most recent ATA guidelines.

As a consequence, between October 2014 and June 2017 only 30 consenting patients were recruited for TL plus IpsiCND-FSE. The C-group was chosen among the more numerous group of patients who underwent TT plus IpsiCND-FSE during the same study period, using a propensity score analysis and matched for age, sex, and tumor size.

We found that FSE correctly identified all the patients with occult central lymph node metastases >2 mm (five in TL-group -16.6%- and six in the C-group -20.0%-, respectively). On the other hand, FSE was not able to identify microscopic (<2 mm) [27] lymph node involvement. However, this last is usually considered of little clinical interest and patients may be managed as at low risk if the lymph node micrometastases are <5 [1].

Of note, eight patients in TL-group underwent completion thyroidectomy + completion CND after FH, seven due to multifocal disease (five of these patients had concomitant micromestastes also -≤2 mm- at FH), one due to extracapsular invasion. After reoperation, FH showed multifocal bilateral PTC in one case and no pathologic findings in the remaining seven cases. Overall 13 out of 30 (43.3%) TL-group patients underwent bilateral surgery and in 5/13 (38.4%) cases no reoperation was required since FSE showed occult lymph node metastases and completion thyroidectomy and bilateral CND was accomplished during the same surgical procedure.

However, we have to say that the choice of completing thyroidectomy and CND in those eight patients who were re-operated after obtaining the FH report was based on histological characteristics historically considered as “high-risk features” such as multifocality and extracapsular invasion [1, 13, 35]. On the other hand, indications for completion surgery after TL are not unequivocal and the recent published 8th edition of TNM [36] have raised further concerns since patients with evidence of minimal extracapsular invasion at FH were now staged as pT1 and the completion thyroidectomy is not always mandatory. In our series five patients (16%) in the TL-Group and six patients (20%) in the C-Group were postoperatively classified as pT3 according to 7th edition of TNM. Unfortunately, also an accurate preoperative US evaluation could overlook microscopic extracapsular invasion but the clinical impact of these finding is to date matter of debate [36].

Although guidelines have evolved to allow for less aggressive treatment options (active surveillance or TL without the need for RAI or TSH suppressive therapy) these recommendations are not only based on limited or conflicting data but are also applicable to properly selected patients. For these reasons many clinicians (both endocrinologists and surgeons) and patients still strongly favor TT as safe and definitive treatment for low risk PTC. In other words to date, despite guidelines recommendations, the best initial extent of thyroidectomy for low risk PTC is still debated. In spite of these controversies pN1 status is still considered a high-risk characteristic requiring a surgical strategy more than a TL. In our series all the patients, initially scheduled TL, with occult macro N1a disease were detected intraoperatively after FSE on IpsiCND and the completion thyroidectomy and CND were performed during the same operation. Larger prospective studies are necessary to further validate the present study but the results seem encouraging.

In addition, the results of the presented study demonstrated that for those PTC patients who are clinically eligible for TL the upstaging rate is equivalent to that obtainable with a more aggressive approach (i.e., TT plus prophylactic CND). Indeed, no difference regarding the accuracy in staging was observed between TL-group and C-group and, at least at a mean follow-up of 22.2 ± 10.6 (range 8–43) months and no recurrences was observed in both groups. Obviously, the accurate knowledge of the nodal status is of utmost importance to correctly stratify PTC patients, especially if a conservative approach is chosen.

Finally, despite for those patients who require bilateral thyroid resection and complete central compartment clearance the complication rate were similar in both groups, overall in the TL-group the transient hypocalcemia rate was significantly reduced, as expected, since <50% of the patients underwent bilateral thyroid resection.

It is evident that the major limitations of the present study are the non-randomization and the small number of patients included. In spite of these limitations, our study has the merit of comparing two groups of T1 PTC, well matched by propensity score analysis, operated on, and followed-up with the same therapeutic and clinical protocol at the same institution. Furthermore, at our knowledge, this is the first report of this intraoperative decision-making approach for preoperative known T1 clinically unifocal, intrathyroidal, cN0 PTC.

In conclusion IpsiCND-FSE allows for an accurate risk stratification of PTC patients eligible for TL and for personalization of the surgical approach. IpsiCND-FSE can safely and correctly identify during the operation patients who benefit from TT plus bilateral CND, reducing the need of a second-step completion procedure and, theoretically, the risk of recurrence.
